# Interaction of LRRK2 with kinase and GTPase signaling cascades

**DOI:** 10.3389/fnmol.2014.00064

**Published:** 2014-07-09

**Authors:** Joon Y. Boon, Julien Dusonchet, Chelsea Trengrove, Benjamin Wolozin

**Affiliations:** ^1^Department of Pharmacology and Experimental Therapeutics, Boston University School of MedicineBoston, MA, USA; ^2^Neurology, Boston University School of MedicineBoston, MA, USA

**Keywords:** GTPase, kinase, trafficking, cytoskeleton, actin, autophagy, cell death, dopamine

## Abstract

LRRK2 is a protein that interacts with a plethora of signaling molecules, but the complexity of LRRK2 function presents a challenge for understanding the role of LRRK2 in the pathophysiology of Parkinson’s disease (PD). Studies of LRRK2 using over-expression in transgenic mice have been disappointing, however, studies using invertebrate systems have yielded a much clearer picture, with clear effects of LRRK2 expression, knockdown or deletion in *Caenorhabditis elegans* and *Drosophila* on modulation of survival of dopaminergic neurons. Recent studies have begun to focus attention on particular signaling cascades that are a target of LRRK2 function. LRRK2 interacts with members of the mitogen activated protein kinase (MAPK) pathway and might regulate the pathway action by acting as a scaffold that directs the location of MAPK pathway activity, without strongly affecting the amount of MAPK pathway activity. Binding to GTPases, GTPase-activating proteins and GTPase exchange factors are another strong theme in LRRK2 biology, with LRRK2 binding to rac1, cdc42, rab5, rab7L1, endoA, RGS2, ArfGAP1, and ArhGEF7. All of these molecules appear to feed into a function output for LRRK2 that modulates cytoskeletal outgrowth and vesicular dynamics, including autophagy. These functions likely impact modulation of α-synuclein aggregation and associated toxicity eliciting the disease processes that we term PD.

## INTRODUCTION

Parkinson’s disease (PD) is the most common age-related motor disorder (Hsu et al., 2010a; Ferree et al., 2012). Accumulation of aggregated α-synuclein to form Lewy bodies is a neuropathological hallmark for PD (Lees et al., 2009). Mutations in leucine-rich repeat kinase 2, LRRK2, gene are common genetic determinants of PD, with at least 20 different mutations identified to date causing late-onset, familial autosomal-dominant PD (Gasser et al., 2011; Greene, 2012). The most prevalent amino acid substitution mutation in LRRK2, G2019S, has been found in 1–2% of sporadic PD cases; with sporadic PD and LRRK2-associated PD being clinically and neurochemically indistinguishable (Healy et al., 2008). These all indicate that LRRK2 has an important role in the pathogenesis of PD.

LRRK2 is a large ubiquitous cytoplasmic protein consisting of 2527 amino acids with multiple functional domains (Cookson, 2010). LRRK2 has a homolog in mammals, termed LRRK1; LRRK1 and 2 expression appear to be complementary, with LRRK2 expression peaking in development and decreases rapidly after birth, while LRRK2 expression increases postnatally (Simon-Sanchez et al., 2006). The LRRK2 protein consists of kinase, Ras-of-complex proteins (ROCs) GTPase, C-terminal of ROC (COR), leucine-rich repeat, ankyrin and WD40 domains (Cookson, 2010). The two catalytic domains are the kinase and ROC GTPase domains. The kinase domain has the highest sequence homology to mitogen-activated protein kinase kinase kinase (MKKK)/MLKs (mixed lineage kinases) and receptor-interacting protein (RIP) kinase families. The most common mutation in LRRK2 associated with PD is the G2019S mutation located in the kinase domain of LRRK2 (Wszolek et al., 2004; Zimprich et al., 2004). Other common amino acid substitution include R1441C/G/H in the Roc GTPase domain and Y1699C in the COR domain (Taylor et al., 2006). The pathological mutations G2019S enhances the kinase activity without affecting the GTPase activity of LRRK2; whereas R1441C/G/H and Y1699C affect GTPase activity of LRRK2 by impairing GTP hydrolysis and has inconsistent effects on its kinase activity (West et al., 2005; Greggio et al., 2006; Ito et al., 2007; Lewis et al., 2007; Li et al., 2007). However, the underlying mechanisms linking such mutations to pathology still remain unclear.

## LRRK2 MODULATES SURVIVAL OF DOPAMINERGIC NEURONS, AUTOPHAGY, AND NEURONAL OUTGROWTH

Studies of LRRK2 using over-expression in transgenic mice have been disappointing, with few studies showing consistent effects occurring upon over-expression of wildtype or mutant LRRK2 (Dawson et al., 2010). Knockout of LRRK2 in the mouse yielded a strong phenotype in the kidney pointing to a role for LRRK2 in autophagy, however, no clear differences in the brain; this suggests that mammalian LRRK2 *in vivo* models do a better job of modeling cellular function/dysfunction rather than providing a more general model of PD (Tong et al., 2010). In contrast, over-expression studies using invertebrate systems have yielded much clearer results. Orthologs of LRRK2 also exist in *Drosophila* and Nematodes, although these species exhibit just one form of LRRK, referred to as lrk-1 (Marin, 2008). The invertebrate LRRK2 orthologs have kinase, COR and ROC domains that are homologous to mammalian LRRK2, and the lrk-1 protein appears to subsume the functions of both LRRK1 and LRRK2 (Marin, 2008; Saha et al., 2009). Studies from several groups examining the effects of LRRK2 expression, knockdown or deletion in *Caenorhabditis elegans* and *Drosophila* all show strong effects (Liu et al., 2008; Wang et al., 2008; Saha et al., 2009; Samann et al., 2009; Venderova et al., 2009; Yao et al., 2010; Yuan et al., 2011). The results appear to differ somewhat based on the system, but the effects are strong in all cases. Studies in our laboratory indicate that expressing WT LRRK2 promotes survival of dopaminergic neurons in response rotenone treatment, while mutant LRRK2 (G2019S or R1441C) enhances loss of dopaminergic neurons relative to WT (Saha et al., 2009). Another important aspect of our observations, which becomes important when considering studies of LRRK2 more generally, is that G2019S also potentiates loss of neuronal processes relative to WT LRRK2. Because nematodes lack endogenous α-synuclein, we recently crossed the LRRK2 lines to a line expressing α-synuclein in dopaminergic neurons. Interestingly, current studies with a new nematode model in our laboratory shows that both WT and G2019S LRRK2 potentiate age-related loss of dopaminergic neurons (and of their processes), suggesting that understanding the interaction between LRRK2 and α-synuclein is important for modeling the pathophysiology of PD. Studies from the Chen laboratory using a different *C. elegans* model (without incorporation of α-synuclein) show a deleterious effect of WT and G2019S LRRK2, while studies using *Drosophila* indicate that LRRK2 potentiates degeneration of retinal cells and loss of dopaminergic neurons (Liu et al., 2008; Yao et al., 2010). Thus, the specific results depend on the particular model used, but it is clear that invertebrate models are very sensitive to modulation of LRRK2 levels, and mutations in LRRK2 potentiate degeneration of dopaminergic neurons. While mouse models are not particularly sensitive to LRRK2 over-expression, knockout of LRRK2 appears to potentiate autophagic deficits in the kidney, which is a site that normally exhibits strong expression of LRRK2. This provides evidence that the effects observed with invertebrate systems have strong relevance to the mammalian system.

Studies using primary rodent neurons grown in culture have yielded much stronger results than the transgenic mouse models. These studies consistently indicate that expression of mutant LRRK2 potentiates neurodegeneration, and reduces neurite outgrowth (Smith et al., 2005; Chan et al., 2011; Skibinski et al., 2014). Interestingly, acute expression of WT LRRK2 appears to be beneficial, which parallels observations from our studies in *C. elegans*. One study, using in utero electroporation of LRRK2 in the rodent brain also observed detrimental effects of G2019S LRRK2 and beneficial effects of WT LRRK2, suggesting that acute expression of LRRK2 in the rodent brain might produce clearer results than chronic over-expression, such as occurs with transgenic mice (Macleod et al., 2006). Taken together, these results indicate that mutant LRRK2 is detrimental; the effects of WT LRRK2 are less clear, with some data suggesting that WT LRRK2 is beneficial, and other data suggesting that over-expression can be mildly detrimental. In general, most data suggest that loss of LRRK2 expression is detrimental.

## LRRK2 REGULATES MAP KINASE SIGNALING PATHWAYS

Incorporating research from multiple different venues suggests roles for LRRK2 in two differing cellular networks. Prior studies from our laboratory, and subsequent studies from the Kahle laboratory indicate that LRRK2 interacts with the mitogen activated protein kinase (MAPK) pathways (Carballo-Carbajal et al., 2010; Hsu et al., 2010a,b). The MAPK pathway is a signaling network that begins with membrane signaling and extends through multiple successive kinases leading ultimately to phosphorylation of transcription factors that act on gene expression (Yang et al., 2013). Receptor signaling frequently begins with MAP Kinase kinase kinases (MAPKKKs, also termed mixed lineage kinases, MLKs), which are enzymes often act to initiate signaling cascades that ultimately lead to transcriptional regulation (Yang et al., 2013). The MLK family of kinases activates the c-jun NH2-terminal kinase (JNK) pathway by phosphorylating MKK4 and -7 and activates the p38 pathway by phosphorylating MKK3 and -6 (Gallo and Johnson, 2002; Wang et al., 2004). These stress kinase complexes required scaffold proteins in the regulation of their subcellular localization. In particular for the MAP kinase signaling cascades, the JNK-interacting proteins (JIPs) are the group of scaffold proteins in such regulation (Kelkar et al., 2005; Whitmarsh, 2006). Hence, these MKKs are then recruited into a multi-protein complex by scaffold proteins JIPs (Habig et al., 2013). JIP1-3 regulates the specificity and localization of the JNK pathway and JIP4 is a specific scaffolding protein for the p38 pathway (Verhey et al., 2001; Kelkar et al., 2005; Whitmarsh, 2006).

Multiple studies show that LRRK2 interacts with the MAPK pathway and has a kinase domain that is homologous to MAPKKK/MLKs and RIPs (West et al., 2005; Greggio and Cookson, 2009; Carballo-Carbajal et al., 2010; Hsu et al., 2010a,b). However, these same studies suggest that LRRK2 is not a strong activator of the pathway. Our laboratory has shown that LRRK2 binds MKK6, -3, and -7 in HEK293FT (Gloeckner et al., 2009; Hsu et al., 2010a). Binding of LRRK2 to MKK6, -3, and -7, activates the p38 and JNK pathway, but the amount of activation is strikingly modest (Hsu et al., 2010a). Receptor activation of the MAPK pathway produces many fold increases in the activities of the downstream kinases, but over-expressing LRRK2 increases activities of the kinases and downstream transcription factors by 60% or less (Hsu et al., 2010a). Studies from the Kahle laboratory showed similarly low levels of kinase activation upon LRRK2 over-expression (Carballo-Carbajal et al., 2010). These studies showed that LRRK2 upregulates alpha-synuclein transcription in parallel with MEK/ERK activation. This induction of transcriptional upregulation of alpha-synuclein was suppressed by treatment with the selective MAPK/ERK kinase inhibitor, U0126 (Carballo-Carbajal et al., 2010). Disease linked mutations increased activation by only an additional 25–30%. Similar results were observed upon analysis of JIP proteins, which act as scaffolds for MAPKs, and function in transport of MAPKs (Hsu et al., 2010b). We have also shown that LRRK2 binds to JIP1-4 and is associated with increased levels of total JIP1, -3, -4, oligomeric JIP, and ubiquitinated JIP. In addition, G2019S, R1441C and I2020T (but not Y1699C) mutations in LRRK2 increased binding with MKK6 and also levels of JIP4 (Hsu et al., 2010b). The stimulation of JIP oligomerization was particularly striking effect observed upon co-expression of LRRK2 with JIPs.

Despite the relatively modest effect of LRRK2 on the MAPK cascades, LRRK2 produces a robust effect *in vivo*, and the MAPKs appear to be necessary for this function. Our *in vivo* studies in *C. elegans* showed robust protection against mitochondrial stress that was induced by LRRK2 expression. This protection was dependent on the action of MKK6 or p38 because RNAi knockdown of endogenous orthologs of MKK6 or P38 (sek-1 and pmk-1) or deletion of sek-1 in *C. elegans* abolish neuroprotection by LRRK2 (Hsu et al., 2010a). The MAPK cascade also appears to modulate the effects of LRRK2 on autophagy (Plowey et al., 2008). Using redox proteomics, we also demonstrated that LRRK2 regulates proteins associated with the lysosome, including ATPVA6 (Di Domenico et al., 2012). Application of a MAPKK (MEK) inhibitor blocked the effects of LRRK2 on autophagy and on neurite shortening (Plowey et al., 2008). Thus, interactions between LRRK2 and members of the MAPK pathway cause significant physiological effects despite causing only modest changes in phosphorylation.

The discordance between the modest effects of LRRK2 on activation of the MAPK pathway and the strong effects of LRRK2 in protecting dopaminergic neurons in the nematode, or the strong effect of LRRK2 on JIP oligomerization demands a novel model to explain LRRK2 function. One possibility is that LRRK2 acts as a scaffold that directs the location of MAPK pathway activity, without strongly affecting the amount of MAPK pathway activity. Location is as important to enzymatic action as amount of activity. JIPs for instance, are thought to function in vesicular trafficking. If LRRK2 also exhibited a role in vesicular trafficking, then it could impact on neuronal function by directing particular enzymes toward trafficked vesicles, rather than explicitly activating the MAPK pathway. Such a role would also be consistent with other studies suggesting a function for LRRK2 in regulating small GTPases associated with vesicles.

## LRRK2 REGULATES MANY OTHER SIGNALING CASCADES

Recent transcriptome and proteome studies emphasize the diverse number of pathways regulated by LRRK2. Studies by the Dawson group recently highlighted regulation of translational functions by LRRK2, showing that LRRK2 binds to and phosphorylates the ribosomal protein RPS15 (Martin et al., 2014). This observation fits well with other studies showing roles for LRRK2 as a negative mediator of miR mediated translational repression (Gehrke et al., 2010). The regulatory network that we developed also identifies RPS15 as a primary member of the LRRK2 regulatory network, interacting with LRRK2 in a pathway that includes the ADP-ribose polymerase TNKS (Dusonchet et al., 2014). The Cookson group also recently published a proteomic study of LRRK2, which identified cyclin-G associated kinase (GAK) as a strong binding protein (Beilina et al., 2014). GAK is the third strongest genetic risk factor for PD, after synuclein and tau (Pankratz et al., 2009; Dumitriu et al., 2011).

Other studies identify LRRK2 as a negative regulator of PKA signaling, acting through direct interaction with PKAR11B, modulating neuronal development and function, by influencing dopamine signaling and synaptogenesis (Parisiadou et al., 2014). The R1441C LRRK2 mutation exhibits similar neuronal effects on LRRK2 knockout mice, causing dopamine signaling impairments as well as neurite retraction via enhanced PKA activation. PKA also regulates LRRK2 by phosphorylating S1444 in the ROC domain; 14-3-3 then binds to the phospho-serine 1444 and inhibits LRRK2 kinase activity (Muda et al., 2014). Binding of 14-3-3 to different phospho-epitopes (Ser-910/935) appears to stabilize LRRK2, while reduced binding destabilizes LRRK2 and causes aggregation of LRRK2 to form cytoplasmic inclusions (Dzamko et al., 2010). Identification of targets of LRRK2 kinase activity remains unclear. Studies have identified numerous putative substrates, including MAPK, 4E-BP, and Tau (Imai et al., 2008; Berwick and Harvey, 2011; Bailey et al., 2013).

Although LRRK2 has gained the most attention for its putative role in modulating dopamine function, LRRK2 also exerts regulation over immune responses. LRRK2 inhibits the transcription factor NFAT, which plays important roles in immune function as well as inflammatory bowel disease (Liu et al., 2011). Upon over-expression of LRRK2, NFAT remains cytosolic, and is unable to translocate into the nucleus, which suppresses its transcriptional activity (Liu et al., 2011). The MyD88 pathway is an additional pathway that is associated with the role of LRRK2 in immune function. Upon inflammation, TLR signaling via the MyD88 pathway leads to phosphorylation of LRRK2, implicating LRRK2 in macrophage biology (Dzamko et al., 2012).

## LRRK2 REGULATES SMALL GTPases

A repeating theme in LRRK2 biology is its interactions with small GTPases. The ROC domain in LRRK2 is striking because it forms homo- and hetero- dimers with LRRK2 and LRRK2, respectively, *in vitro* (Shin et al., 2008; Hsu et al., 2010a; Kumar et al., 2010). The tendency of the LRRK2 ROC domain to dimerize appears to belie a broader biological characteristic, which is an ability to bind multiple small GTPases. As described below, we have shown that LRRK2 binds the small GTPase, rac1 (Chan et al., 2011). This observation parallels other studies showing that LRRK2 binds to or is functionally dependent on other small GTPases, such as rab5, rab7L1 and endoA (Matta et al., 2012; Beilina et al., 2014). Additionally, LRRK2 binds multiple GTP modulating proteins (described in following sections). In the case of rac1, the interactions of LRRK2 appears to regulate the site of action of the small GTPase, and leads to strong effects on the cytoskeleton, and for arfGEF7, they regulate the growth cone (Habig et al., 2013).

The interaction between LRRK2 and rac1 was observed by co-immunoprecipitation. LRRK2 could immunoprecipitate with rac1 upon over-expression in cell lines (HEK293FT cells), as well as from endogenous human brain striatum; in contrast binding to Cdc42 was weak and binding to RhoA was not apparent (Chan et al., 2011). Specificity of the interaction was shown by selective precipitation of rac1, without precipitation of the other classic Rho GTPases – Cdc42 and RhoA, although another study did find evidence for interaction of LRRK2 with cdc42 (Chan et al., 2011; Habig et al., 2013). The interaction between LRRK2 and rac1 occurs through the ROC–COR kinase domains. Co-expressing LRRK2 and rac1 enhanced rac1 activity by increasing binding of rac1 to p21-activated kinase, which in turn modulates actin cytoskeletal dynamics. LRRK2 with inactivated kinase or GTPase domains does not activate rac1 (Chan et al., 2011). LRRK2 does not increase membrane-bound rac1, but it significantly changes the cellular localization of rac1, causing polarization, which is further augmented when LRRK2 is co-expressed with constitutively active rac1. G2019S and R1441C LRRK2 mutations decrease rac1 binding; whereas Y1699C and I2020T increase rac1 binding.

Rac1 is known to play an important role in actin cytoskeleton remodeling that is required for the maintenance of neurite morphology (Chan et al., 2011). The interaction between LRRK2 and rac1 results in distinct effects associated with changes in the actin cytoskeleton. Previous studies have shown that G2019S induces neurite retraction in both *in vitro* and *in vivo* studies through the MAPK signaling pathway and such pathology precedes dopaminergic neuronal death by apoptosis (Liou et al., 2008; Plowey et al., 2008; Parisiadou et al., 2009; Carballo-Carbajal et al., 2010; Hsu et al., 2010a,b). In SH-SY5Y cells, co-expression of rac1 and G2019S has shown to rescue neurite retraction induced by G2019S. These studies suggest that mutations in LRRK2 can lead to a decrease in activation of rac1, which causes disassembly of actin filaments leading to neurite retraction (Chan et al., 2011).

## SYSTEMS BIOLOGY PROVIDES A COMPREHENSIVE ASSESSMENT OF CELLULAR PATHWAYS INTERACTING WITH LRRK2

The studies showing LRRK2 interacting with many varied cellular proteins present a challenge for understanding its function. LRRK2 has been suggested to bind to many different proteins, including moesin, tubulin, MKK3, 6 and 7, JIP1, 3, and 4, arfGAP1, arhGEF7, endoA, cyclin-GAK, rab5, rab7L1, 14-3-3, (Imai et al., 2008; Shin et al., 2008; Ko et al., 2009; Sancho et al., 2009; Gehrke et al., 2010; Hsu et al., 2010a,b; Kumar et al., 2010; Nichols et al., 2010; Chan et al., 2011; Matta et al., 2012; Stafa et al., 2012; Habig et al., 2013; Beilina et al., 2014). LRRK2 has a regulatory role in a wide variety of biological processes; such as, protein translations, cytoskeletal processes, vesicular dynamics, neurite extension, mitochondrial function, endoplasmic reticulum function, and autophagy. Broad proteomics studies point to interactions with multiple new proteins, as well as regulation of many mitochondrial and lysosomal proteins (Di Domenico et al., 2012; Beilina et al., 2014). The multiple functions and pathways associated with LRRK2 suggest a complex role for LRRK2 in neuronal biology. We have approached this question by using systems biology to create a regulatory network that outlines the multiplicity of functional interactions of LRRK2 with its partners.

We employed an *in silico* approach to elucidate the gene regulatory network linked to LRRK2 (Dusonchet et al., 2014). This approach used a network algorithm, termed the context likelihood of relatedness (CLR; Faith et al., 2007). This algorithm is designed to analyze state-dependent genome-wide expression data based on the degree of synchrony in variation of transcript levels among samples. Thus, transcripts whose expression varies in concert with LRRK2 transcripts are deemed neighbors. We analyzed both brain and white blood cells; use of white blood cells diversified the pathway representation away from a predominance of genes linked to cell death pathways, presumably because white blood cells are not post-mortem tissues. The LRRK2-centered association sub-network identified many known interactors, as well as many novel linked pathways (Dusonchet et al., 2014). For instance, network proteins that have been previously associated with LRRK2 are present in this network, including DJ-1, PINK1, MKK7, and JIP1 (Ho et al., 2009; Sancho et al., 2009; Carballo-Carbajal et al., 2010; Hsu et al., 2010a,b; Ferree et al., 2012; Dusonchet et al., 2014). The network also identified many novel networks linked to LRRK2. Subsequent studies validated the putative roles of these interacting proteins using knockdown studies in *C. elegans*.

Previous work from the laboratory established that LRRK2 expression protects dopaminergic neurons against degeneration induced by the mitochondrial toxin, rotenone. We tested the action of the putative network partners in LRRK2 mediated protection of dopaminergic neurons, and identified about 280 genes whose knockdown modified the effects of LRRK2 on dopaminergic neurons. Genes whose knockdown impaired LRRK2 action included genes known to be part of the LRRK2 network (DJ-1, PINK1, MKK7, and JIP1), as well as multiple other genes, including HDAC6, vps34 and unc51, each of which is associated with autophagy (Dusonchet et al., 2014). The connection between LRRK2 and autophagy is consistent with several other prior studies, as well as with studies suggesting a role for LRRK2 in vesicular biology (Biskup et al., 2006; Plowey et al., 2008; Alegre-Abarrategui et al., 2009).

**FIGURE 1:**
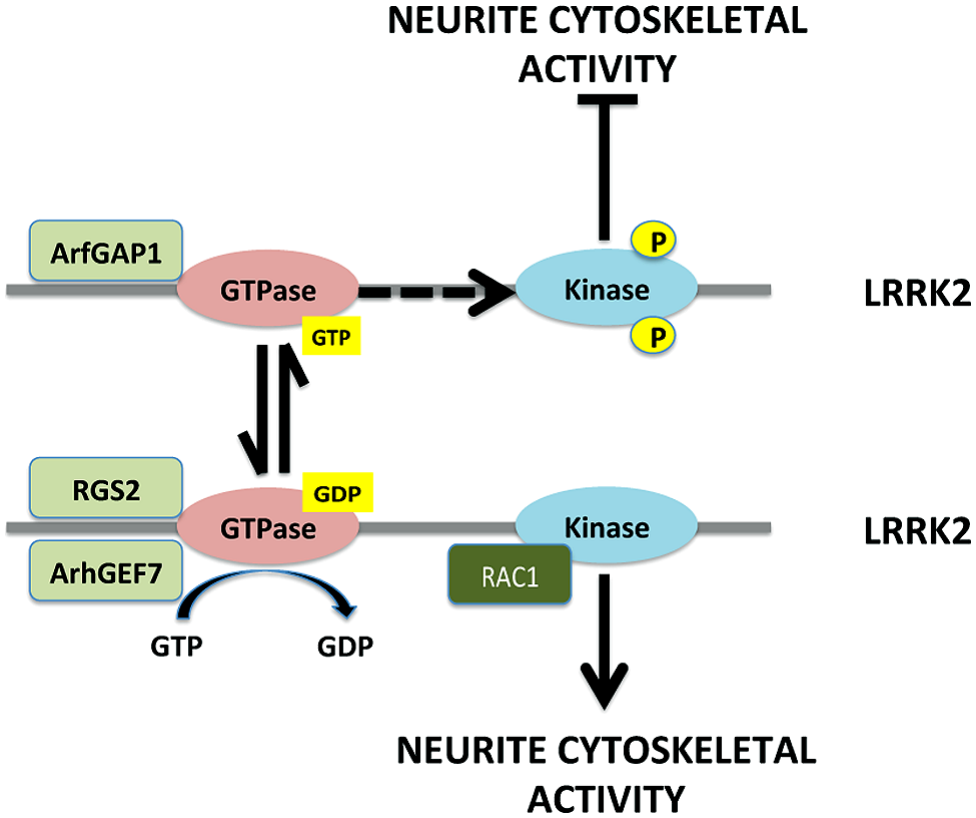
**Regulation of LRRK2 output by GTPase activity, GAPs and GEFs.** RGS2 and ArhGEF7 both bind to LRRK2 and inhibit GTPase activity, which also inhibits LRRK2 kinase activity and appears to direct LRRK2 toward increasing neuronal cytoskeletal activity. Rac1 is a small GTPase known to regulate actin polymerase. Rac1 binds to LRRK2 and might act in concert with LRRK2 to increase cytoskeletal activity. In contrast, binding of ArfGAP1 to LRRK2 increases its autophosphorylation, which appears to inhibit cytoskeletal activity.

One particularly interesting proteins identified through the network analysis was the signaling gene RGS2 (regulator of G protein signaling 2), which encodes for a GTPase-activating protein (GAP), as a statistically significant regulatory “hub” in the pathways linking LRRK2 with DJ-1, PINK1, and Parkin (Dusonchet et al., 2014). RGS2 is also of particular interest because prior studies indicate strong expression in dopaminergic neurons. This positioning of RGS2 as a “hub” for multiple genes linked to PD suggests a key role for RGS2 as a regulator of LRRK2 activity, function and neuronal toxicity (Dusonchet et al., 2014). RGS proteins are a family of proteins characterized by a GAP domain of ~130 amino acids, the RGS domain. Other GAP proteins have also been associated with LRRK2 function, including ArfGAP1 and ArhGEF7 (Stafa et al., 2012; Xiong et al., 2012; Habig et al., 2013). Although GAPs and GTPase exchange factors (GEFs) are classically considered to function in regulating signaling of G-protein coupled receptors, these GAPs exhibit a strong role in regulating LRRK2 GTPase activity. Recombinant RGS2 and ArhGEF7 increase the GTPase activity of immunopurified full-length LRRK2 in a dose-dependent manner *in vitro*. Recombinant RGS2 also inhibits the LRRK2 kinase activity in a dose-dependent manner. However, the concentration of RGS2 required to achieve maximal inhibition of LRRK2 kinase activity, occurs at one tenth of the concentration of RGS2 that is required to stimulate GTPase activity maximally. It is increasingly clear that the output of LRRK2 is modulated by the particular GAP or GEF bound to it. All of the GAPs and GEFs appear to increase LRRK2 GTPase activity, but RGS2 and ArhGEF7 reduce LRRK2 kinase activity, while ArfGAP1 increases LRRK2 autophosphorylation (Stafa et al., 2012; Habig et al., 2013; Dusonchet et al., 2014). This suggests that the output from the GTPase domain is determine, in part, by the particular GAP or GEF with which it is associated (**Figure 1**).

## GAPs AND GEFs MODULATE CYTOSKELETAL EFFECTS OF LRRK2

A putative role for LRRK2 in regulating cytoskeletal function consistently appears in the literature. LRRK2 has been observed to regulate neurite outgrowth, tubulin and microtubules, trafficking proteins and actin (Hsu et al., 2010b; Chan et al., 2011; Caesar et al., 2013; Law et al., 2014). Our studies suggest that RGS2 inhibits the tendency of LRRK2 (WT and G2019S) to reduce length and complexity of neuronal processes in primary neurons (**Figure 1**; Dusonchet et al., 2014). Co-expression of RGS2 with LRRK2 significantly protects against G2019S LRRK2-induced neurite shortening, although RGS2 alone has no effect on axonal length. ArhGEF7 also increases LRRK2 GTPase activity but induces LRRK2 to increase neuronal arborization, length and growth cone formation (**Figure 1**; Habig et al., 2013). In contrast, knockdown of ArfGAP1 protects against G2019S LRRK2-induced neurite shortening (**Figure 1**; Stafa et al., 2012). ArhGEF7 acts through the actin cytoskeleton, which raises the possibility that it might act in tandem with rac1 and cdc42, both of which bind LRRK2 (Chan et al., 2011; Habig et al., 2013). These data suggest that small GTPases and their regulatory proteins act to regulate the actions of LRRK2 on the cytoskeleton and neurite outgrowth (**Figure 1**). Redundant actions of RGS2 and ArhGEF7 toward neurite cytoskeletal activity seems unlikely, which raises the possibility that RGS2 and ArhGEF7 exhibit divergent effects on LRRK2 when examining other functions, such as vesicular biology.

## CONCLUSION

LRRK2 is a large molecule with many molecular interactions. Multiple studies have focused on its kinase activity, with the resulting identification of LRRK2 inhibitors that might have therapeutic benefit. The function of LRRK2 kinase activity remains unclear, with LRRK2 itself being the most robust substrate identified to date. Meanwhile, LRRK2 has been shown to interact with many different proteins, suggesting that important function of LRRK2 might lie in domains linked indirectly or not at all to its kinase function. A recent study suggests that an important action of phosphorylation is regulation of LRRK2 degradation, which would imply that one must look beyond kinase function to understand the role of LRRK2 in the neuron (Skibinski et al., 2014). The need to look beyond kinase function is emphasized by examination of the actions of GAPs and GEFs on LRRK2, where proteins that exhibit seemingly similar actions toward LRRK2 kinase activity elicit opposite actions on regulation of neurite outgrowth and cytoskeletal function (**Figure 1**).

Studies from our laboratory and other laboratories have identified numerous other signaling molecules that interact with LRRK2. The interactions of LRRK2 are clearly pleiotropic, and vary depending on the cell type and the function being investigated. Despite this complexity, several distinct themes are evolving, and these themes potentially have important implications for our understanding of the pathophysiology of PD. One theme that arises is the role of LRRK2 in regulating signaling cascades, such as the MAPK cascade. It is clear that LRRK2 acts in a manner that is different than classic modulators of the MAPK cascade, because it does not directly stimulate phosphorylation of MAP Kinases. The large size of LRRK2 enables it to bind multiple different proteins, and potentially bring them together as a signaling scaffold, analogous to JIPs.

A general theme occurring throughout the field of PD is regulation of vesicular transport. The vast majority of genes associated with PD have some function related to membranes and vesicles. α-Synuclein is thought to regulate production of small vesicles by promoting membrane curvature (Perlmutter et al., 2009; Varkey et al., 2010; Ducas and Rhoades, 2012). LRRK2 also shows strong interactions with membranes and is thought to modulate autophagy, which also involves vesicular dynamics. PINK1, parkin, and HTRA2 are all proteins that regulate mitochondrial function, possibly including mitophagy (Plun-Favreau et al., 2007; Narendra et al., 2010). ATP13A2 and GBA are both associated with lysosomes (Mazzulli et al., 2011; Usenovic et al., 2012). The consistent appearance of vesicular biology in the pathophysiology of PD suggests that interactions of LRRK2 with vesicles are likely to contribute to its mechanism of disease pathogenesis. Future studies will need to specifically investigate how signaling pathways regulate the interactions of LRRK2 with membranous organelles, such as autophagosomes. In this context, the preliminary data showing roles for the MAPK cascade in regulating the interactions of LRRK2 with the autophagic system are intriguing (Plowey et al., 2008).

The complexity of LRRK2 signaling provide insight into the nature of pathology associated with LRRK2-mediated disease. Most cases with LRRK2 mutations exhibit α-synuclein pathology, such as Lewy bodies, but some cases exhibit tau pathology (Devine and Lewis, 2008). This pathological heterogeneity might derive from the impact of different disease processes on LRRK2 biology, with some signaling cascades (such as MAPKs) promoting tau pathology and other signaling cascades (such as vesicular biology) promoting α-synuclein pathology.

## AUTHOR CONTRIBUTIONS

Joon Y. Boon drafted the manuscript, Chelsea Trengrove helped with revisions, and Benjamin Wolozin edited the manuscript. Julien Dusonchet provided important concepts for the manuscript.

## Conflict of Interest Statement

Benjamin Wolozin has a conflict of interest based on ownership of equity in Aquinnah Pharmaceuticals, Inc., which is a company focusing on neurodegenerative diseases. Joon Y. Boon, Chelsea Trengrove, and Julien Dusonchet have no conflicts of interest.
